# Early Extracellular ATP Signaling in *Arabidopsis* Root Epidermis: A Multi-Conductance Process

**DOI:** 10.3389/fpls.2019.01064

**Published:** 2019-09-04

**Authors:** Limin Wang, Gary Stacey, Nathalie Leblanc-Fournier, Valérie Legué, Bruno Moulia, Julia M. Davies

**Affiliations:** ^1^Department of Plant Sciences, University of Cambridge, Cambridge, United Kingdom; ^2^Divisions of Plant Science and Biochemistry, University of Missouri, Columbia, MO, United States; ^3^Université Clermont Auvergne, INRA, PIAF, Clermont-Ferrand, France

**Keywords:** ATP, anion, channel, DORN1, P2K1, root epidermis, ROS

## Abstract

Adenosine 5′-triphosphate (ATP) is an important extracellular signaling agent, operating in growth regulation, stomatal conductance, and wound response. With the first receptor for extracellular ATP now identified in plants (P2K1/DORN1) and a plasma membrane NADPH oxidase revealed as its target, the search continues for the components of the signaling cascades they command. The *Arabidopsis* root elongation zone epidermal plasma membrane has recently been shown to contain cation transport pathways (channel conductances) that operate downstream of P2K1 and could contribute to extracellular ATP (eATP) signaling. Here, patch clamp electrophysiology has been used to delineate two further conductances from the root elongation zone epidermal plasma membrane that respond to eATP, including one that would permit chloride transport. This perspective addresses how these conductances compare to those previously characterized in roots and how they might operate together to enable early events in eATP signaling, including elevation of cytosolic-free calcium as a second messenger. The role of the reactive oxygen species (ROS) that could arise from eATP’s activation of NADPH oxidases is considered in a qualitative model that also considers the regulation of plasma membrane potential by the concerted action of the various cation and anion conductances. The molecular identities of the channel conductances in eATP signaling remain enigmatic but may yet be found in the multigene families of glutamate receptor-like channels, cyclic nucleotide-gated channels, annexins, and aluminum-activated malate transporters.

## Introduction

Adenosine 5′-triphosphate (ATP) is well known as an essential cellular energy source. However, the recognition of ATP as an extracellular signaling agent in plants is becoming more widespread (Clark and Roux, 2018). Extracellular ATP (eATP) has been shown to modulate growth and development, particularly of pollen and root hairs (Roux and Steinebrunner, 2007; Clark et al., 2010; Wu et al., 2018). It is abundant at the apex of growing roots and root hairs in a range of plants (Kim et al., 2006) and is involved in root gravitropism and root curling (Tang et al., 2003; Yang et al., 2015). eATP can also regulate stomatal movement (Clark et al., 2011; Hao et al., 2012; Wang et al., 2014; Chen et al., 2017). Activation of plant stress responses by eATP, notably wounding responses, may be through second messengers such as nitric oxide, reactive oxygen species (ROS), and cytosolic-free calcium ([Ca^2+^]_cyt_) (Demidchik et al., 2003a; Song et al., 2006; Foresi et al., 2007; Torres et al., 2008; Wu et al., 2008; Demidchik et al., 2009; Choi et al., 2014). A key advance in the field comes from the identification of the first angiosperm eATP receptor, P2K1 (DORN1, does not respond to nucleotides 1), in *Arabidopsis thaliana*. The P2K1 nomenclature is preferred since this aligns the plant work with the greater body of animal literature focused on the P2X and P2Y families of purinergic receptors. The P2K1 plasma membrane (PM) receptor kinase commands increases in ROS and [Ca^2+^]_cyt_ by eATP that operate in seedling wound transcriptional response and regulation of stomatal aperture (Choi et al., 2014; Chen et al., 2017).

Ion fluxes across the PM are likely to be critical components of early eATP signal cascades, particularly in the generation of a [Ca^2+^]_cyt_ signal. The majority of research to date on eATP-induced ion fluxes has been on root cells, which have proven to be sensitive and experimentally tractable. eATP has been found to depolarize (i.e., make more positive) the PM potential of growing *Arabidopsis* root hairs (Lew and Dearnaley, 2000), indicating cation influx/anion efflux. It has also been observed to affect root PM Ca^2+^, K^+^, and Na^+^ fluxes (Dark et al., 2011; Demidchik et al., 2011; Lang et al., 2014; Zhao et al., 2016). Moreover, the Ca^2+^ and K^+^ fluxes in response to eATP vary spatially along the root (measured using an extracellular, self-referencing ion-selective microelectrode; Dark et al., 2011; Demidchik et al., 2011). The *Arabidopsis* elongation zone epidermis proved more sensitive to eATP than the mature zone, also sustaining greater net Ca^2+^ influx and K^+^ efflux (Dark et al., 2011; Demidchik et al., 2011). Such Ca^2+^ influx across the PM could relate to eATP-induced [Ca^2+^]_cyt_ increase as a second messenger. eATP has now been shown to elevate root [Ca^2+^]_cyt_, measured using the luminometric reporter aequorin and fluorescence resonance energy transfer (FRET)-based reporters such as YC3.6 (Demidchik et al., 2003a; Demidchik et al., 2009; Tanaka et al., 2010; Loro et al., 2012; Behera et al., 2018). Blocking putative PM Ca^2+^ influx channel proteins with lanthanides or chelating extracellular Ca^2+^ can prevent eATP-induced [Ca^2+^]_cyt_ elevation (Demidchik et al., 2003a; Demidchik et al., 2009; Behera et al., 2018), implicating such passive transporters in the generation of the [Ca^2+^]_cyt_ signal.

Patch clamp electrophysiology has been applied successfully to resolve eATP-activated PM Ca^2+^ influx channels in *Arabidopsis* root cells. Mature epidermal cells have a hyperpolarization activated calcium channel (HACC) conductance that is further activated by eATP (Demidchik et al., 2009). Similar HACC conductances activated by eATP have since been identified at the guard cell and pollen PM (Wang et al., 2014; Wu et al., 2018). In root epidermis, the HACC may lie downstream of the PM RBOHC NADPH oxidase isoform. This HACC may contribute to the net Ca^2+^ influx reported for this root zone (Demidchik et al., 2009; Shang et al., 2009; Dark et al., 2011; Demidchik et al., 2011). Patch clamping has also implicated the heterotrimeric G protein α subunit in eATP activation of the PM HACC conductance of apical root cells (Zhu et al., 2017). Furthermore, patch clamping of elongation zone epidermal PM has revealed a small HACC-like conductance (which also permits K^+^ influx) and a K^+^ efflux conductance (in 44 out of 113 protoplasts) that not only are activated by eATP but also lie downstream of P2K1 (Wang et al., 2018). The K^+^ efflux pathway resembles a depolarization-activated nonselective cation channel conductance (NSCC; Wang et al., 2018). It is feasible that these could contribute to the Ca^2+^ influx and K^+^ efflux evoked by eATP in the elongation zone epidermis (Demidchik et al., 2009; Dark et al., 2011; Demidchik et al., 2011). Thus, so far, little is known about the regulation of plant PM channels by eATP. Based on further patch-clamp studies here of PM conductances from the root elongation zone epidermis, early ionic events in response to eATP (narrowed down to the level of ion channel conductance) are revealed in this perspective.

## Diverse Conductances in the Plasma Membrane of *Arabidopsis* Root Epidermis

**Figure 1:**
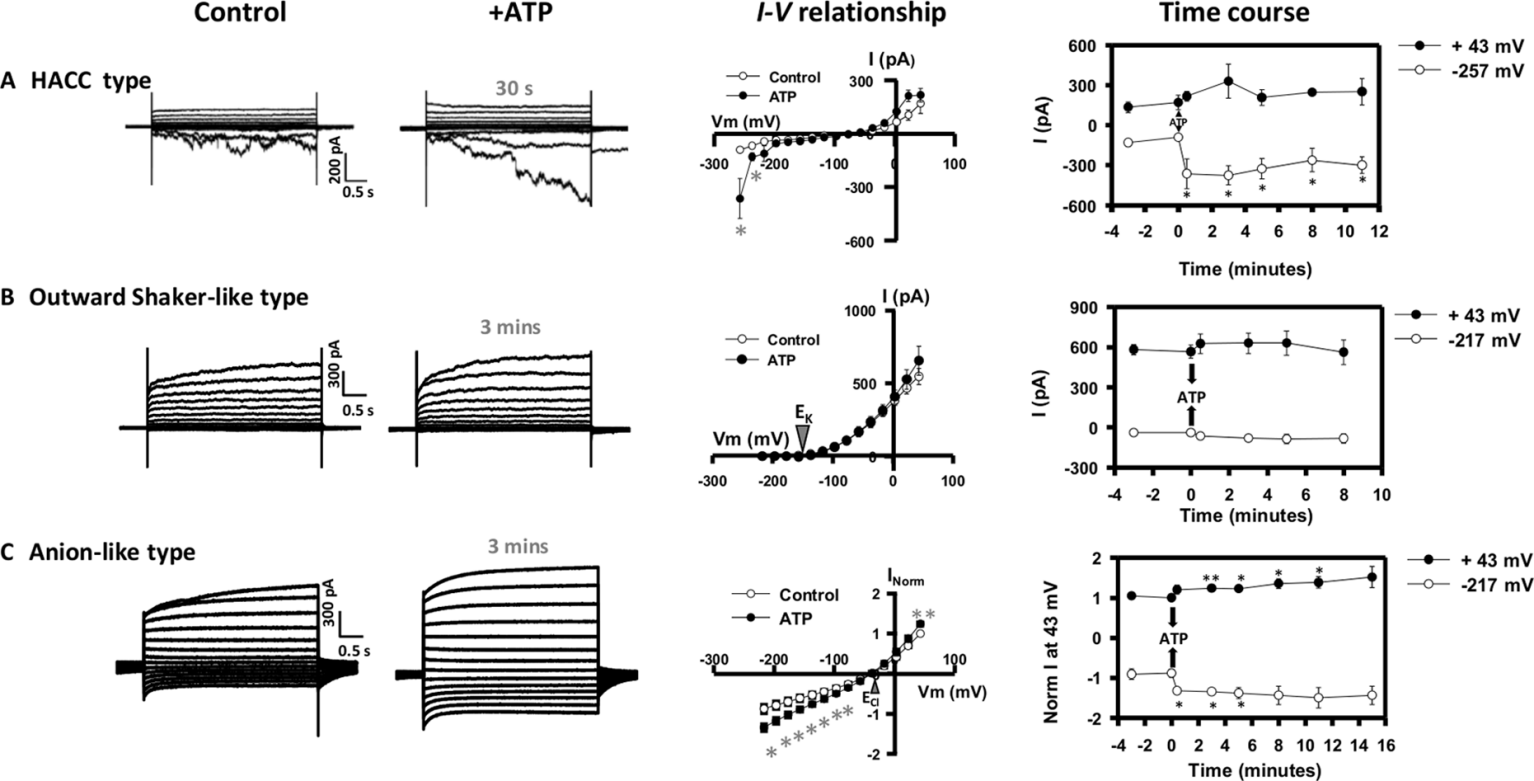
Effect of extracellular adenosine 5′-triphosphate (eATP) on diverse plasma membrane (PM) conductances from the root elongation zone epidermis. Protoplasts were isolated and used in whole-cell patch clamp recordings as described previously (Wang et al., 2018). Origin of the protoplasts was confirmed with the N9093 epidermal-specific green fluorescent protein (GFP) reporter line (Diatloff et al., 2004). This configuration measures populations of channels. Plasma membrane potential was held at −137 mV prior to a stepwise voltage protocol of 20 mV increments. Whole-cell currents were recorded in a bath solution containing (mM) 20 CaCl2, 0.1 KCl, and 5 MES-Tris, at pH 5.6. Pipette solution comprised (mM) 40 K-gluconate, 10 KCl, 0.4 CaCl2, 1 BAPTA, and 2 MES-Tris, at pH 7.2. Osmolarity of both solutions was adjusted to 280–290 mOsm with d-sorbitol. Representative current traces of **(A)** the hyperpolarization activated calcium channel (HACC) conductance, **(B)** the outward Shaker-like conductance, and **(C)** the anion conductance under control and eATP conditions (300 µM) are shown in the left panel. Corresponding mean I–V relationships for control (○) and eATP (•) treatments are shown in the central panel with time of treatment indicated. The right panel presents the time course of eATP-activated outward currents at +43 mV (•) and inward currents at −257 mV/−217 mV (○) for each type of conductance. Data are mean ± SE (n = 4 in **A**, 5 in **B**, and 4 in **C**). Negative current is net cation influx or anion efflux. Positive current is net cation efflux or anion influx. Asterisks denote significant difference from control. *p < 0.05, **p < 0.01 (Student’s *t*-test).

A range of Ca^2+^ channels, K^+^ channels, NSCC, and anion channels have been identified previously in *Arabidopsis* root epidermal PM through patch clamping (e.g., Demidchik et al., 2002; Foreman et al., 2003; Pilot et al., 2003; Diatloff et al., 2004; Demidchik et al., 2007; Demidchik et al., 2009; Hedrich et al., 2012; Laohavisit et al., 2012; Demidchik et al., 2014; Makavitskaya et al., 2018). Using the same experimental conditions as our previous study (which identified the eATP-activated small HACC-like and K^+^ efflux conductances; Wang et al., 2018), 26 out of 113 protoplasts from the elongation zone epidermis were found to have a large time-dependent HACC conductance (Véry and Davies, 2000) under control conditions, which was accompanied by an instantaneous outward current at depolarized voltages (**Figure 1A**). eATP increased HACC currents rapidly (within a minute) after treatment, and activation lasted for at least 10 min (**Figure 1A**). This was a similar time course to the eATP-activated HACC from mature epidermal protoplasts, in which activation persisted for up to 20 min (Demidchik et al., 2009). NaCl (600 µM, the control for the Na-ATP salt) did not cause HACC activation (**Figure S1**). eATP-induced HACC inward currents were blocked by the lanthanide cation channel blocker Gd^3+^, indicating cation permeability (**Figure S2A**). Qualitatively, the eATP-activated HACC resembled those found in *Arabidopsis* root tip cell PM, *Vicia faba* guard cell PM, and tobacco pollen PM (Wang et al., 2014; Zhu et al., 2017; Wu et al., 2018).

Protoplasts (11 out of 113) also presented a conductance dominated by a non-linear outward current that activated around the equilibrium potential for K^+^ [E_K_ annotated on the current voltage (*I–V*) graph in **Figure 1B**]. This resembled previously characterized Shaker outward K^+^ channel conductances (Gaymard et al., 1998; Ache et al., 2000; Hosy et al., 2003; Li et al., 2016) and would mediate K^+^ efflux from the cytosol. Similar to the plant Shaker outward K^+^ channels reported so far (Gaymard et al., 1998; Ache et al., 2000; Hosy et al., 2003; Li et al., 2016; Wang et al., 2019), this conductance was inhibited by external application of the classical K^+^ channel blocker, tetraethylammonium (TEA) (**Figure S2B**). This Shaker-like outward conductance was not significantly affected by eATP (**Figure 1B**). This distinguishes the conductance from the eATP-activated NSCC K^+^ efflux conductance found by Wang et al. (2018). Additionally, the time constant of activation at 23 mV (185.3 ± SE 22.7; *n* = 6) of the Shaker-like outward conductance is twofold slower than the NSCC outward conductance, suggesting that they are distinct conductances.

An anion conductance was evident in 12 protoplasts. This reversed close to E_Cl_ (**Figure 1C**), indicating an anion (Cl^−^) permeability. The *I–V* relationships for control and eATP trials of the individual protoplasts tested are shown in **Figure S3**. There was variation in the magnitude of current, and statistical analysis of the eATP effect was after normalization (Maierhofer et al., 2014). Qualitatively, this conductance resembles a root epidermal PM conductance that permits ascorbate efflux (Makavitskaya et al., 2018), and the mild deactivation at negative voltages resembles that of the wheat Al^3+^-activated ALMT1 anion channel (Zhang et al., 2008). Anion fluxes (especially Cl^−^ fluxes) in eATP signaling are poorly documented, possibly due to the methodological limitations of using self-referencing ion-selective electrodes (Shabala et al., 2013; Pottosin et al., 2018). This anion conductance would permit anion efflux at hyperpolarized voltage and anion influx at depolarized voltage. Anion influx responded rapidly (within a minute) to eATP, while efflux was significantly increased after 3 min and was significant for several minutes after (**Figure 1C**). The eATP-activated conductance was insensitive to Gd^3+^ (**Figure S2C**), further supporting its identity as an anion conductance. This conductance may be relevant to the effects of eATP on membrane voltage. Overall, of the 113 protoplasts studied, the most frequently occurring conductances were the small HACC-like conductance (which also permits K^+^ influx) and the K^+^ efflux conductance reported by Wang et al. (2018). The remaining 20 protoplasts of the 113 that were not described here did not display a clear conductance type.

## Multiple Conductances Could Operate in Root Epidermal eATP Signaling

**Figure 2:**
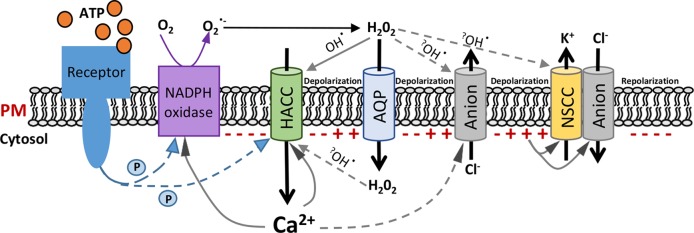
Schematic of a hypothetical pathway of extracellular adenosine 5′-triphosphate (eATP)-activated conductances in root epidermal plasma membrane. Hypothetical model integrating the eATP-induced plasma membrane (PM) conductances from this study and previous findings referenced in the main text. The signal cascade is presented from left to right, starting with eATP perception by the receptor. Polarity of the PM potential at the cytosolic face is represented by “−” or “+”. Phosphorylation is indicated by “P”. An early event would be Ca^2+^ influx through hyperpolarization-activated Ca^2+^ channels (HACCs). Extracellular H_2_O_2_ could enter the cytosol through aquaporins (AQPs). H_2_O_2_ could directly act on ion channels or be converted to hydroxyl radicals (OH•) through Fenton reactions (indicated by question marks). Anion channels would sequentially permit Cl^−^ efflux; then influx and nonselective cation-permeable channels (NSCCs) would facilitate K^+^ efflux. The overall sequence would promote repolarization of the PM potential. Arrows indicate possible activation pathways but do not necessarily imply direct interactions. The dashed arrows are predicted pathways, which are highly recommended to be investigated in future.

Combining this new knowledge of eATP-activated root epidermal conductances with findings from previous studies (Véry and Davies, 2000; Demidchik et al., 2003a; Demidchik et al., 2003b; Demidchik et al., 2007; Demidchik et al., 2009; Shang et al., 2009; Demidchik et al., 2011; Tavares et al., 2011; Choi et al., 2014; Wilkins et al., 2016; Chen et al., 2017; Rodrigues et al., 2017; Gutermuth et al., 2018; Pottosin et al., 2018; Wang et al., 2018) allows generation of a hypothetical and qualitative model of the early steps in eATP signaling in *Arabidopsis* epidermis (**Figure 2**). This presumes that the conductances found to be activated by eATP here and by Wang et al. (2018) would all be present in one cell, despite the varying frequency of occurrence in patched protoplasts. Those frequencies may reflect different levels of cellular maturity at the point of release or perhaps even the PM state (pump state, K^+^ state, or depolarized state; Tyerman et al., 2001) at the initiation of patching. In this model, eATP is expected to modulate the root epidermal PM potential through the regulation of these ion conductances. eATP recognition is postulated to be by the PM receptor P2K1 (Choi et al., 2014; Wang et al., 2018). This could possibly phosphorylate the channels involved here, with the HACC as a prime target. However, in guard cells, P2K1 phosphorylates the respiratory burst oxidase protein D (RBOHD) NADPH oxidase, resulting in elevated production of ROS (Chen et al., 2017). This could also occur in the root epidermis (perhaps even with the RBOHC isoform; Demidchik et al., 2009) as eATP can increase root epidermal cytosolic ROS (mainly H_2_O_2_) within seconds in an RBOH-dependent manner, which in turn activates downstream [Ca^2+^]_cyt_ signaling (Demidchik et al., 2009; Demidchik et al., 2011). It is envisaged that extracellular H_2_O_2_ (as a downstream product of RBOH activity) could enter the cytosol through PM aquaporins, in common with guard cells (Rodrigues et al., 2017). Due to the fast activation found here of the HACC conductance upon eATP addition (**Figure 1A**), this HACC may therefore be directly or indirectly responsive to ROS (**Figure 2**). Which ROS and at which membrane face? Activation of elongation zone epidermal HACC by extracellular H_2_O_2_ has been found, but the time course of activation was not reported (Demidchik et al., 2007). Entry of H_2_O_2_ into the cytosol could also produce intracellular hydroxyl radicals [formed through a Cu^+^ catalyst in the Fenton reaction (Richards et al., 2015) to activate Ca^2+^ influx (Rodrigo-Moreno et al., 2013)]. HACC activation in this cell type by extracellular hydroxyl radicals occurs in a few minutes (Foreman et al., 2003) and also occurs in mature epidermis (time course not reported; Laohavisit et al., 2012). All scenarios assume that ROS could be generated under patch clamp conditions. Supporting this, eATP activation of the mature epidermis HACC in patch clamp was lost in the *rbohc* loss-of-function mutant and prevented in wild type by the reductant dithiothreitol, suggesting that ROS production is possible (Demidchik et al., 2009). Also, activation of guard cell PM HACC by eATP was prevented by DPI (diphenyleneiodonium), an inhibitor of flavoproteins including NADPH oxidases, placing the HACC downstream of such enzymes (Wang et al., 2014).

As Ca^2+^ is transported into the cytosol, it could lead to a depolarization of the root epidermal PM and possibly have a positive feedback effect on the RBOH (through EF hands) and the HACC (Wilkins et al., 2016). It has been shown previously that increased [Ca^2+^]_cyt_ shifts the HACC activation threshold to depolarized voltage and increases current magnitude (Véry and Davies, 2000; Demidchik et al., 2002). Then, a subsequent Cl^−^ release at a more depolarized voltage through the eATP-activated anion conductance (**Figure 1C**) could deepen the PM depolarization (**Figure 2**). It may also be that Cl^−^ efflux through the anion conductance is stimulated by the increased [Ca^2+^]_cyt_. The precedent for this comes from the *Arabidopsis* pollen tube apical PM, where hyperpolarization-induced [Ca^2+^]_cyt_ increase causes increased Cl^−^ efflux (Tavares et al., 2011), possibly through Ca^2+^-dependent protein kinases (Gutermuth et al., 2018). Another stimulator could be eATP-induced ROS (Kim et al., 2006; Demidchik et al., 2009). Indeed, it has been reported that extracellular hydroxyl radicals could induce efflux of cytosolic anions from barley elongation zone epidermal protoplasts, which could contribute to root PM depolarization (Pottosin et al., 2018). If the eATP-activated anion conductance found here were capable of releasing ascorbate to the extracellular PM face (Makavitskaya et al., 2018), it could even promote ascorbate-fueled extracellular hydroxyl radical production (Richards et al., 2015; Makavitskaya et al., 2018).

After sufficient depolarization, the activation of Cl^−^ *influx* through the anion conductance (**Figure 1C**) and K^+^ efflux through the NSCC-like conductance (Wang et al., 2018) would increase. The latter was found only to be significant after 8 min of exposure to eATP (Wang et al., 2018) and may well be a late event. Qualitatively, this NSCC-like conductance resembles an elongation zone PM NSCC conductance found to be activated by extracellular hydroxyl radicals (Demidchik et al., 2003b). It may be that hydroxyl radicals are involved in eATP signaling. Alternatively, as high extracellular H_2_O_2_ inhibits K^+^ efflux by the PM NSCC (Demidchik et al., 2003b), late activation of the NSCC-like conductance could reflect the lowering of H_2_O_2_ concentration at the extracellular PM face. The induction of cation efflux and anion influx upon longer ATP treatment (>3 min) could finally repolarize the PM of the root epidermis. Although the NSCC-like conductance found by Wang et al. (2018) is proposed to participate in the PM repolarization in the present model (**Figure 2**), a potential role for the Shaker-like outward conductance (shown in **Figure 1B**) cannot be excluded. When PM repolarizes to a certain voltage, passing the activation potential of the NSCC-like conductance, the Shaker-like outward conductance might contribute (probably after 8 min) to continuing the PM repolarization, thus eventually hyperpolarizing the plasma membrane.

## Future Directions

While eATP has been shown to depolarize the PM (Lew and Dearnaley, 2000), showing the dependence on P2K1 would be critical to start verifying this model. P2K1 has been shown to be required for the eATP-activated root epidermis PM HACC-like and NSCC-like conductances (Wang et al., 2018). Whether P2K1 (or an as yet unknown receptor; Clark and Roux, 2018) governs the eATP-induced HACC and anion currents remains, however, to be elucidated. The relationship between P2K1 and RBOHs in the root epidermis also needs to be tested, as does the possible role of ROS in activating the conductances found in the present study. It has been reported that H_2_O_2_ induces reactive carbonyl species (RCS) and that these significantly inhibit K^+^ inward channels in guard cell PM (Islam et al., 2016). It would be interesting to test root epidermis overexpressing 2-alkenal reductase (an RCS scavenger; Islam et al., 2016) to see whether eATP signaling would normally result in inhibition of K^+^ inward channels through RCS production.

Searching for the molecular identities of these root epidermal conductances in eATP signaling is imperative. Patch-clamp analyses of cyclic nucleotide-gated channel (CNGC) mutants suggested that CNGC2, CNGC4, CNGC5, and CNGC6 from *Arabidopsis* could contribute to HACC conductances (Ali et al., 2007; Gao et al., 2012; Wang et al., 2013; Tian et al., 2019). The CNGC family has also been proposed to encode NSCC (Köhler et al., 1999; Jammes et al., 2011; Demidchik, 2014). So far, *Arabidopsis* CNGC14 has been discounted as a contributor to eATP-induced [Ca^2+^]_cyt_ elevation in roots (Shih et al., 2015). *Arabidopsis* CNGC20 may be a candidate if eATP were to promote production of intracellular ROS, as this channel subunit has been found at the PM (Fischer et al., 2013) and may have an intracellular copper-binding site to permit Fenton generation of hydroxyl radicals for its own activation (Demidchik et al., 2014). In mature epidermis and root hairs, the hydroxyl radical-activated HACC is entirely reliant on Annexin1 (Laohavisit et al., 2012), raising the possibility of this protein’s involvement in younger cells. In addition to the CNGC family and annexins, the glutamate receptor-like (GLR) family provides other candidates for HACCs and NSCC (Roy et al., 2008; Tapken and Hollmann, 2008; Swarbreck et al., 2013; Toyota et al., 2018). *Arabidopsis* GLR3.3 and GLR3.6 operate in wound-induced leaf [Ca^2+^]_cyt_ increase (Vincent et al., 2017) and so would be prime candidates. For the Shaker-like K^+^ efflux conductance that appeared insensitive to eATP, it could be shaped by the guard cell outward rectifier K (GORK) channel, since this is expressed in the root epidermis and has been characterized as a root K^+^ outward channel in *Arabidopsis* (Ivashikina et al., 2001; Demidchik, 2014). Moreover, it can contribute to root cell PM hyperpolarization (Planes et al., 2014), consistent with a role in restoring the PM voltage at the end of eATP signaling. However, root PM GORK releases K^+^ in response to extracellular hydroxyl radicals (Demidchik et al., 2010), which is at odds with the production of this ROS in the current model. The expectation would be for GORK to be activated, but with maximal activation by radicals occurring after 15–20 min (Demidchik et al., 2010); recordings here may not have been long enough. Moreover, GORK is a tightly regulated channel, controlled by its positional clustering (Eisenach et al., 2014), 14-3-3 binding, and [Ca^2+^]_cyt_-dependent phosphorylation status (Van Kleeff et al., 2018), and so other regulatory factors could be at play.

The novel finding here of an eATP-activated anion conductance adds another component to eATP signaling. Plant PM anion fluxes can involve slow-activating and rapid-activating anion channels, provided by members of the slow anion channel-associated (SLAC) and aluminum-activated malate transporter (ALMT) families, respectively (Hedrich et al., 2012). At this point, an ALMT channel appears the most likely candidate for the eATP-activated anion channel, but members of the ATP-binding cassette superfamily should be considered given that mammalian ABC transporters can function as Cl^−^ channels (Anderson et al., 1991). In addition to this perspective on the molecular identities of channels in eATP signaling, it is important to note two other transporters that are omitted from our simplistic model; the PM H^+^-ATPase and Ca^2+^-ATPase. The PM H^+^-ATPase plays a major part in generating the membrane potential, setting the electrochemical driving force for eATP-induced Ca^2+^ influx. AHA2 is the predominant PM H^+^-ATPase in *Arabidopsis* root cells (Falhof et al., 2016). Accordingly, *Arabidopsis* roots lacking the AHA2 isoform have a lower eATP-induced [Ca^2+^]_cyt_ increase than wild type (Haruta and Sussman, 2012). Whether the eATP-induced [Ca^2+^]_cyt_ increase regulates H^+^-ATPase activity remains to be determined. The PM Ca^2+^-ATPases (ACA8 and ACA10) that pump Ca^2+^ out of the cytosol to help end the eATP-induced [Ca^2+^]_cyt_ signal in root cells (Behera et al., 2018) are unlikely to contribute to membrane potential repolarization as such transporters are electroneutral Ca^2+^:2H^+^ exchangers (Luoni et al., 2000). They could, however, contribute to the cytosolic acidification that lags behind the eATP-induced [Ca^2+^]_cyt_ increase in root cells (Behera et al., 2018). This acidification is unlikely to affect the channels mediating Ca^2+^ influx (Behera et al., 2018) but could induce activation of slow anion channels (Colcombet et al., 2005) and the PM H^+^-ATPase (Behera et al., 2018). Whether the activation of PM H^+^-ATPase by the cytosolic acidification (Behera et al., 2018) could help in PM repolarization needs to be addressed.

Overall, further investigation of the functional properties of the root epidermal PM conductances activated by eATP (and other extracellular nucleotides) will be required to make progress in understanding their molecular identities and the downstream signaling pathways.

## Data Availability

The datasets generated for this study are available on request to the corresponding author.

## Author Contributions

LW designed and performed the experiments and then analyzed data. All authors conceived the project. LW and JD jointly conceived and wrote the manuscript with contributions from NL-F, VL, BM, and GS.

## Funding

Results have been achieved within the framework of the third call of the ERA-NET for Coordinating Action in Plant Sciences, with funding from the BBSRC (BB/S004637/1) and the University of Cambridge Newton Trust.

## Conflict of Interest Statement

The authors declare that the research was conducted in the absence of any commercial or financial relationships that could be construed as a potential conflict of interest.
