# Supplementary material for: Early Extracellular ATP Signaling in Arabidopsis Root Epidermis: A Multi-Conductance Process
Source: Front Plant Sci. 2019 Sep 4;10:1064. doi: 10.3389/fpls.2019.01064 (PMC6737080; doi:10.3389/fpls.2019.01064)
Supplement: Supplementary file 1 [file Presentation_1.pptx]

## Slide 1
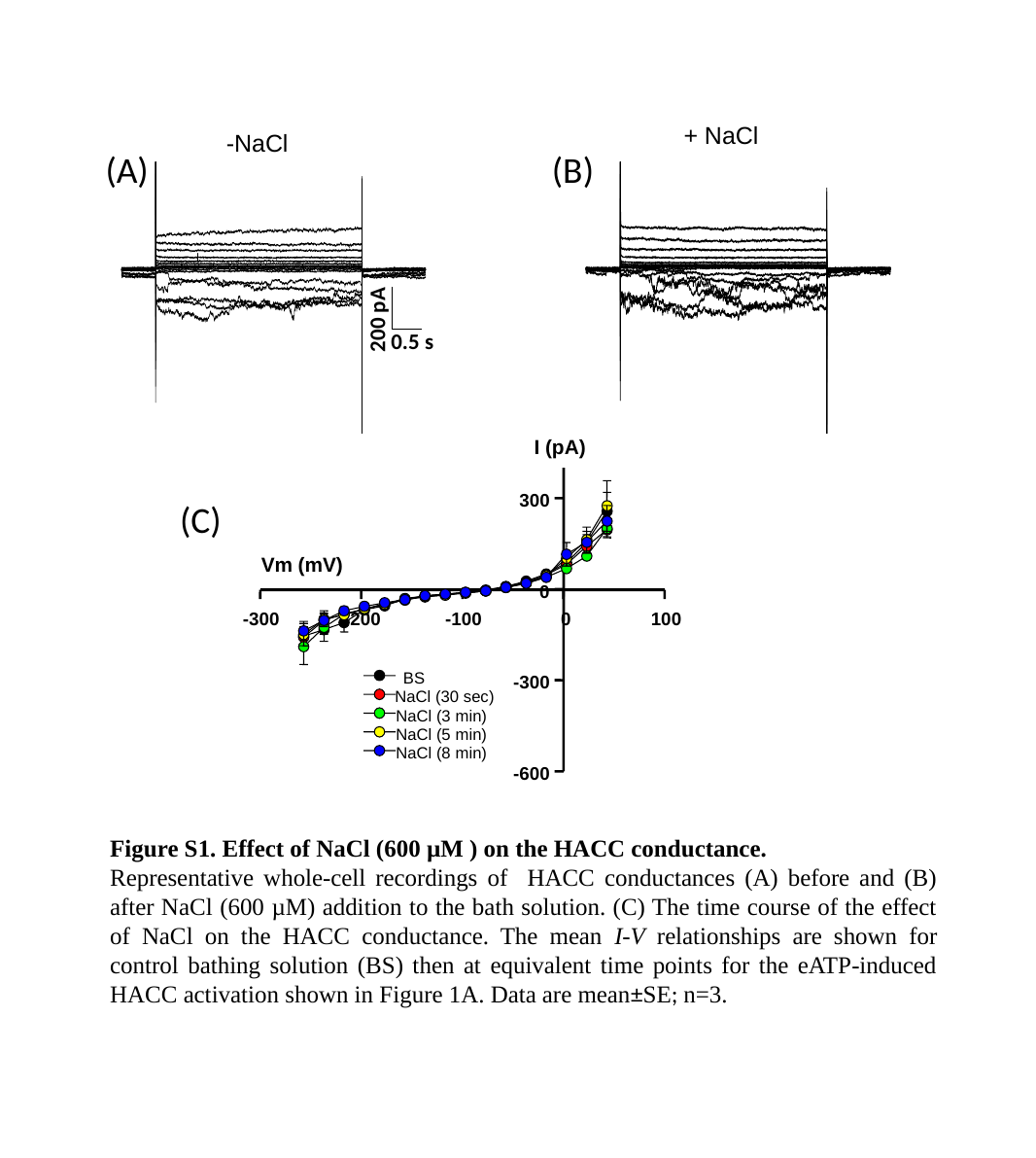

+ NaCl
-NaCl
(A) (B)
 (C)
200 pA
0.5 s
I (pA)
300
Vm (mV)
0
-300
-200
-100
0
100
-300
-600
BS
NaCl (30 sec)
NaCl (3 min)
NaCl (5 min)
NaCl (8 min)
Figure S1. Effect of NaCl (600 µM ) on the HACC conductance.
Representative whole-cell recordings of HACC conductances (A) before and (B) after NaCl (600 µM) addition to the bath solution. (C) The time course of the effect of NaCl on the HACC conductance. The mean I-V relationships are shown for control bathing solution (BS) then at equivalent time points for the eATP-induced HACC activation shown in Figure 1A. Data are mean±SE; n=3.
