# Supplementary material for: Early Extracellular ATP Signaling in Arabidopsis Root Epidermis: A Multi-Conductance Process
Source: Front Plant Sci. 2019 Sep 4;10:1064. doi: 10.3389/fpls.2019.01064 (PMC6737080; doi:10.3389/fpls.2019.01064)
Supplement: Supplementary file 2 [file Presentation_2.pptx]

## Slide 1
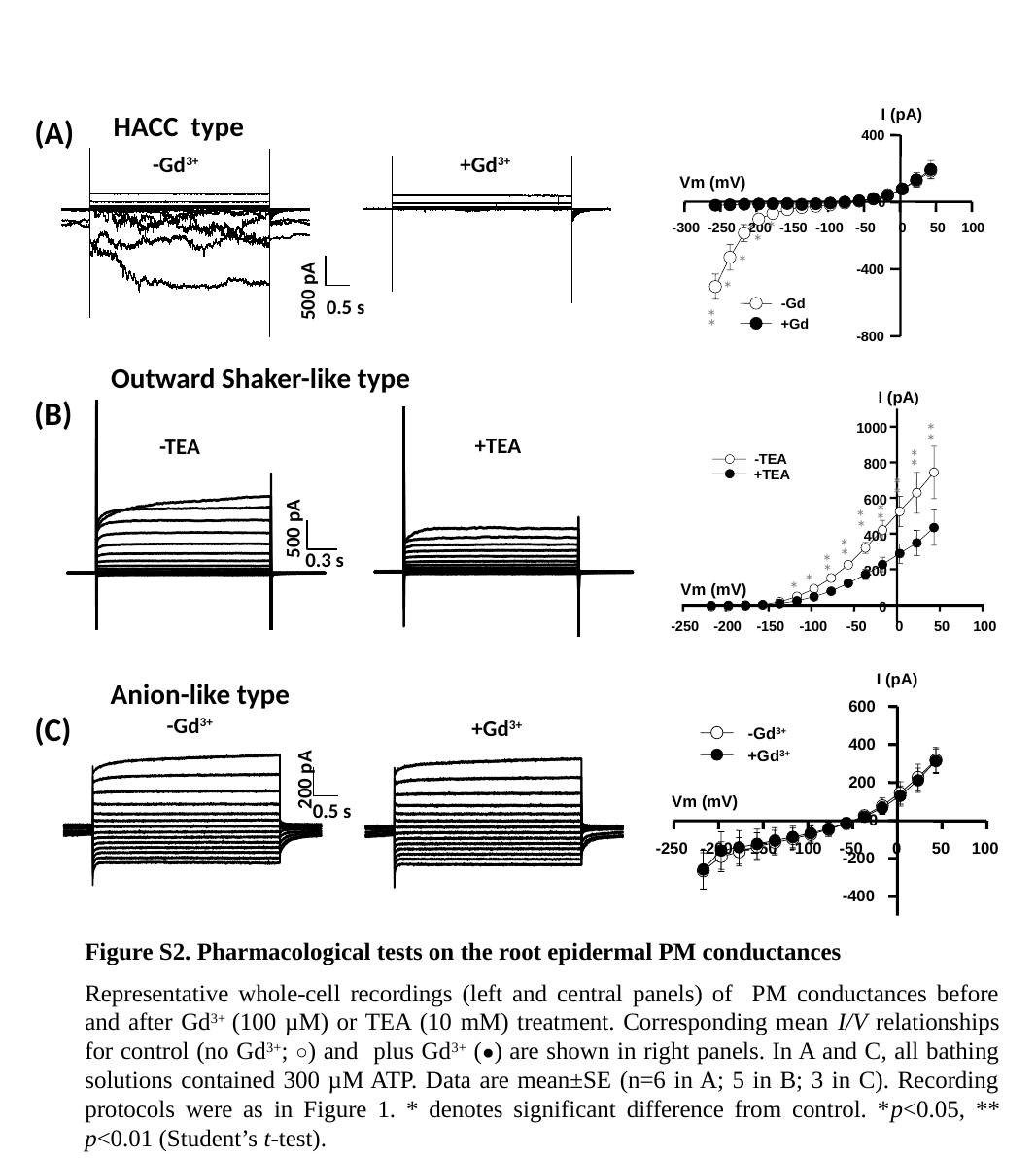

HACC type
I (pA)
400
Vm (mV)
0
-300
-250
-200
-150
-100
-50
0
50
100
-400
-Gd
-800
+Gd
(A)
-Gd3+
+Gd3+
-Gd
+Gd
500 pA
0.5 s
*
*
*
*
-Gd3+
*
*
+Gd3+
 Outward Shaker-like type
(B)
I (pA)
1000
-TEA
800
+TEA
600
400
200
Vm (mV)
0
-250
-200
-150
-100
-50
0
50
100
*
*
+TEA
-TEA
*
*
*
*
*
500 pA
*
*
*
*
*
0.3 s
*
*
*
*
I (pA)
600
-Gd3+
400
+Gd3+
200
Vm (mV)
0
-250
-200
-150
-100
-50
0
50
100
-200
-400
Anion-like type
(C)
-Gd3+
+Gd3+
200 pA
0.5 s
Figure S2. Pharmacological tests on the root epidermal PM conductances
Representative whole-cell recordings (left and central panels) of PM conductances before and after Gd3+ (100 µM) or TEA (10 mM) treatment. Corresponding mean I/V relationships for control (no Gd3+; ○) and plus Gd3+ (●) are shown in right panels. In A and C, all bathing solutions contained 300 µM ATP. Data are mean±SE (n=6 in A; 5 in B; 3 in C). Recording protocols were as in Figure 1. * denotes significant difference from control. *p<0.05, ** p<0.01 (Student’s t-test).
