# Supplementary material for: Early Extracellular ATP Signaling in Arabidopsis Root Epidermis: A Multi-Conductance Process
Source: Front Plant Sci. 2019 Sep 4;10:1064. doi: 10.3389/fpls.2019.01064 (PMC6737080; doi:10.3389/fpls.2019.01064)
Supplement: Supplementary file 3 [file Presentation_3.pptx]

## Slide 1
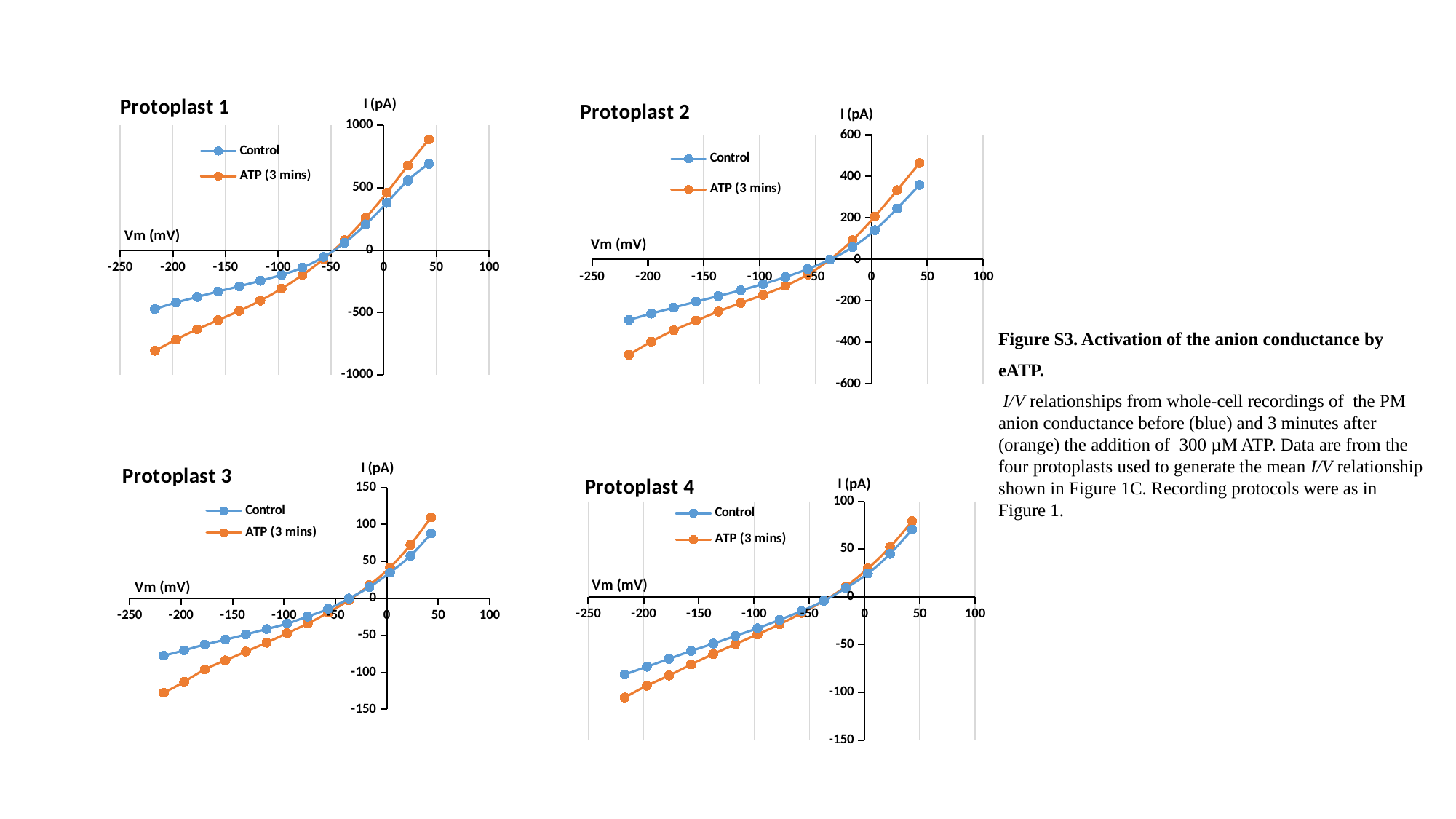

### Chart: Protoplast 1
| Category | | |
|---|---|---|
### Chart: Protoplast 2
| Category | | |
|---|---|---|Figure S3. Activation of the anion conductance by
eATP.
 I/V relationships from whole-cell recordings of the PM
anion conductance before (blue) and 3 minutes after
(orange) the addition of 300 µM ATP. Data are from the
four protoplasts used to generate the mean I/V relationship
shown in Figure 1C. Recording protocols were as in
Figure 1.
### Chart: Protoplast 3
| Category | | |
|---|---|---|
### Chart: Protoplast 4
| Category | | |
|---|---|---|
